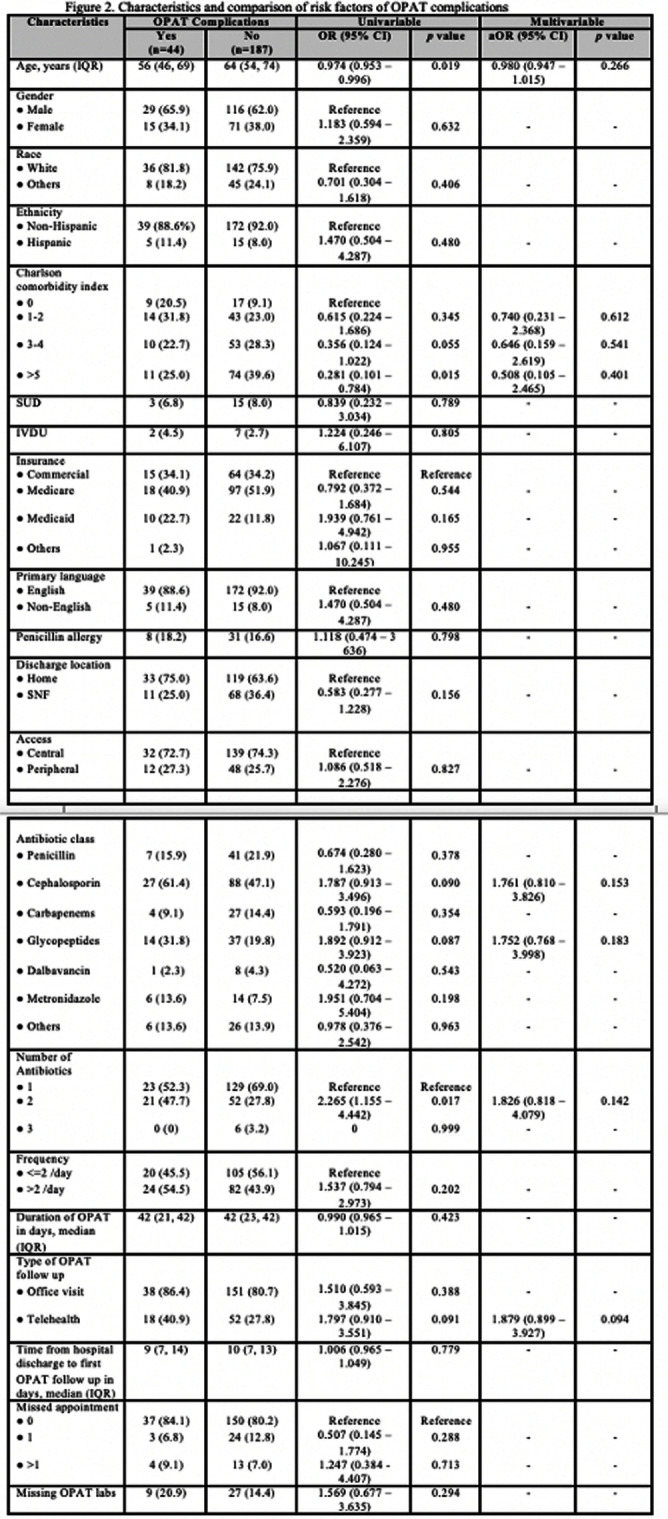# Risk Factors Predicting Complication of OPAT in an Academic Center: A Retrospective Cohort Study

**DOI:** 10.1017/ash.2024.169

**Published:** 2024-09-16

**Authors:** Fang Yu Liu, Kristen McSweeney, Rachel Erdil, Majd Alsoubani, Tine Vindenes, Shira Doron, Kap Sum Foong

**Affiliations:** Tufts Medical Center; Tufts University School of Medicine; Tufts Medicine

## Abstract

**Background:** While Outpatient Parenteral Antibiotic Therapy (OPAT) offers patient convenience and reduced healthcare costs, its increasing utilization has brought various complications to light, including antibiotics-related and line-related OPAT complications. In a large prospective study, 18% of the patients experienced adverse drug events. Another study showed 8.45% of patients had vascular complications. Our study aims to identify clinical predictors associated with OPAT complications. Identifying predictors for suboptimal OPAT outcomes provides an opportunity to intervene, thereby minimizing the risk of OPAT-related complications. **Method:** We conducted a retrospective cohort study at Tufts Medical Center of all adult patients aged ≥18 years discharged on OPAT from April 2022 to October 2022. Demographic, treatment, outcome, and complications data were extracted through chart review. The primary outcome was the proportion and predictors of OPAT complications. The secondary outcomes were OPAT completion rate, 30-day ED visit, and 30-day readmission rates related to OPAT complications. We used univariable and multivariable analyses using logistic regression models for the predictors of OPAT complications. Variables with p5 (OR, 0.281, 95% CI 0.101–0.784), but they were more likely to have received two antibiotics (OR, 2.265; 95% CI 1.155-4.442). However, no significant independent predictor OPAT complications was identified in multivariable regression analysis (Figure 2). OPAT completion rates were lower in patients with complications (59.1% versus 75.4%). The 30-day ED visit and 30-day readmission rates were significantly higher in the complication group (31.8% vs. 0 and 34.1% vs. 2.1%, respectively). **Conclusion:** Our study highlights the significant difference in treatment completion rates and higher incidence of ED visits and readmissions rates among those with OPAT complications. Although specific independent predictor was not identified, the association with multiple antibiotic therapies and telemedicine follow-ups suggests areas for further investigation.

**Figure:**
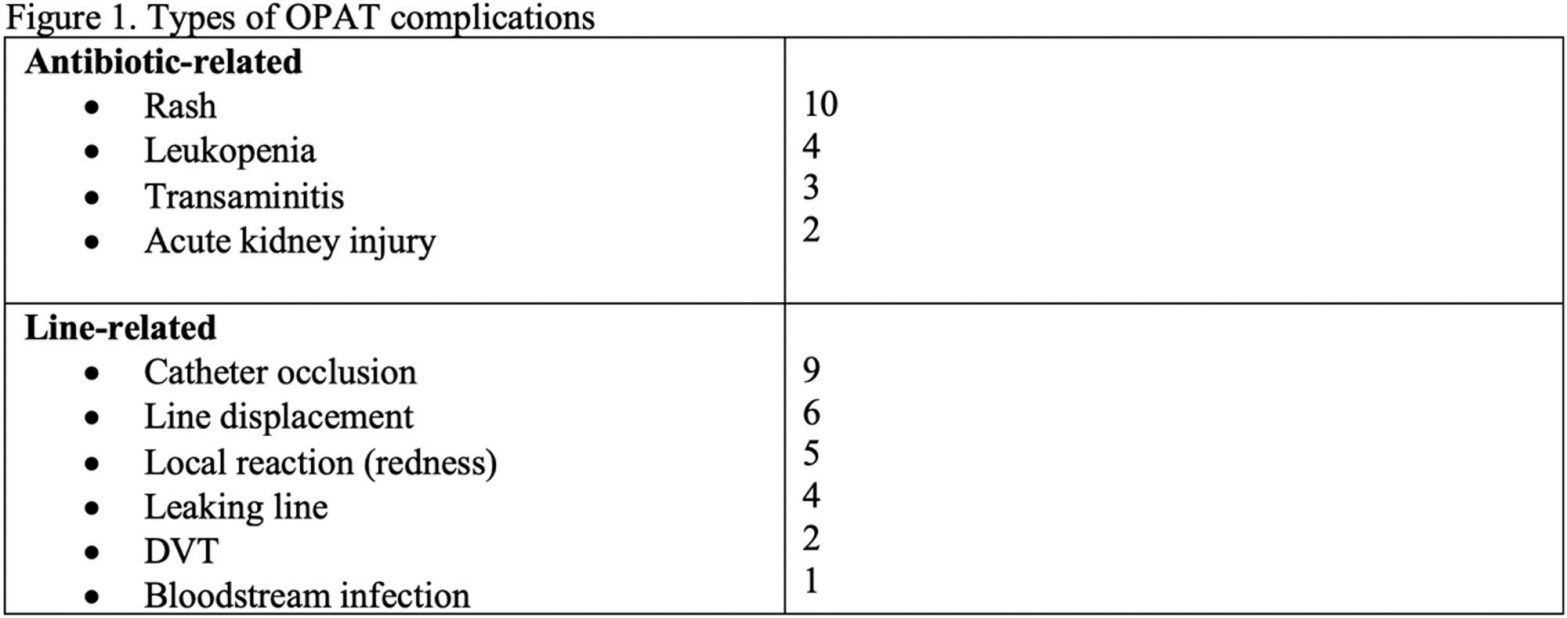

**Figure:**